# Mammary stem cells: expansion and animal productivity

**DOI:** 10.1186/2049-1891-5-36

**Published:** 2014-07-07

**Authors:** Ratan K Choudhary

**Affiliations:** 1 School of Animal Biotechnology, Guru Angad Dev Veterinary and Animal Science University, Ludhiana, Punjab 141004, India

**Keywords:** Hormones, Mammary stem cell, Manipulation, Milk production, Ruminant, Xanthosine

## Abstract

Identification and characterization of mammary stem cells and progenitor cells from dairy animals is important in the understanding of mammogenesis, tissue turnover, lactation persistency and regenerative therapy. It has been realized by many investigators that altered lactation, long dry periods (non-milking period between two consecutive lactation cycles), abrupt cessation of lactation (common in water buffaloes) and disease conditions like mastitis, greatly reduce milk yield thus render huge financial losses within the dairy sector. Cellular manipulation of specialized cell types within the mammary gland, called mammary stem cells (MaSCs)/progenitor cells, might provide potential solutions to these problems and may improve milk production. In addition, MaSCs/progenitor cells could be used in regenerative therapy against tissue damage caused by mastitis. This review discusses methods of MaSC/progenitor cell manipulation and their mechanisms in bovine and caprine animals. Author believes that intervention of MaSCs/progenitor cells could lessen the huge financial losses to the dairy industry globally.

## Introduction

The ultimate goal of mammary gland and mammary stem cell biologists in dairy science is to enhance milk production in lactating dairy animals. Milk production is affected by the number of secretory cells in the mammary alveolar epithelium and the secretory activity per cell. The differentiation status of mammary epithelial cells determines their secretory activity. Poorly differentiated mammary epithelial cells are often non-secretory, whereas intermediate and fully differentiated cells are often secretory in nature [1,2]. Classification of these cells into poor-, intermediate- and fully-differentiated stages, are based on observation of cellular morphology at high magnification for the presence of secretory vacuoles, lipid droplets, nuclear location, cytoplasmic area and cell shape. Although hormones, like estrogen, progesterone and prolactin influence cytological differentiation of these cells but their regeneration depends upon the activity of mammary progenitor cells. Mammary progenitor cells are trans-amplifying cells [3] and are the progeny of mammary stem cells (MaSCs). Reports indicate that MaSCs are multipotent, giving rise to luminal and basal/myoepitheial cell types [4,5]. However, a recent report has indicated MaSCs as lineage-restricted unipotent stem cells in the mouse [6]. This suggests that re-evaluation of MaSCs is required to understand the biology of their cell regulation. For milk-producing dairy animals, more in-depth analysis of the characterization and regulation of MaSCs and progenitor cells is needed before we can understand how to influence cell turnover for increased milk production and tissue homeostasis of the mammary gland. Manipulation of mammary gland development and milk production can be achieved using management of photoperiod, frequent milking, machine milking and bovine somatotrophic (bST) hormone [7,8]. However, manipulation of MaSCs and progenitor cells for increasing milk production is novel and promising, and was first hypothesized by Capuco *et al.*[9,10]. This review deals with relatively recent studies performed towards expansion of MaSCs for determining the impact on milk production. Readers are encouraged to take note of two recent comprehensive review articles on MaSCs in animals of veterinary importance, including a comparative study of post-natal mammary gland development and mammary stem cells in murine and bovine animals [11,12].

## Review

### Mammary stem cells, their identification and characterization

#### MaSCs/progenitor cells

MaSCs are multipotent adult stem cells giving rise to cells of luminal and myoepithelial cell origins. Conventionally, MaSCs are epithelial in origin. In addition to epithelial cells, mammary tissue also comprises cells of mesenchymal origin, including fad pad and connective tissues. Transplantation of dispersed cells into cleared mammary fat pad and clonal expansion of transplanted cell into functional mammary gland, have become gold standard methods to assess the self-renewal property of MaSCs and support the existence MaSC multipotency [13]. In addition, researchers have reported MaSCs as bi-potent [14] and lineage-restricted unipotent stem cells [6]. Indeed, the precise identification and subsequent characterization of MaSCs are conflicting [15] and need to be re-evaluated in the context of their dynamics [16]. Identifying different MaSC subtypes will allow precise targets to be found for optimal manipulation of increased milk production.

One of the main roles of adult stem cells is to proliferate, ensuring organ growth and maintaining tissue homeostasis of the resident organ. During proliferation, stem cells divide symmetrically and when maintaining tissue homeostasis, they divide asymmetrically. Symmetrical division of a stem cell involves mitotic division of the cell into two daughter stem cells or terminally differentiated cells. During asymmetrical division, the stem cell produces one daughter stem cell and one differentiated cell, both cells possessing dissimilar phenotypes. Although, adult stem cells have an unlimited proliferation capacity but divide infrequently *in situ*. Progenitor cells, the progeny of stem cells, have a more limited proliferation capacity in comparison with stem cells, but divide more frequently. Lineage restricted progenitor cells have a tremendous proliferation capacity and are responsible for the generation of differentiated cells to ensure ductal growth, alveolar development and ultimately milk production. The regeneration capacity of MaSCs is evaluated *in vivo* using a transplantation assay in the mammary fat pad of mice that are devoid of mammary epithelium [17,18]. Likewise, the regeneration capacity of progenitor cells is tested *in vitro* by the colony formation assay [19-21].

#### Identification of MaSCs/progenitor cells

Various methods for identification of MaSCs have been performed in different species, as reviewed recently [22,23]. Among these various methods for enriching the MaSC population, utilization of cell surface marker expression is the most common. This method has been used to successfully identify MaSCs in various species including human [24,25], murine [18,26] and bovine [27].

BrdU (bromodeoxyuridine) label-retaining epithelial cells (LRECs) are stem cells identified in various organs, including murine and bovine mammary glands [28,29]. LRECs do not express estrogen and progesterone receptors (ER^-^ and PR^-^ cells), similar to mammary stem cells identified by multiparameter cell sorting in mice [30]. Detailed investigation of LRECs from heifer mammary glands has demonstrated their transcriptome profile that was harvested from the basal layer (hypothesized location of MaSCs) and embedded layer of mammary epithelium layers [31]. Basal layer LRECs were enriched with stem cell transcripts, and therefore were characteristic of stem cells. Likewise, LRECs from the embedded layer were enriched with a few stem cell transcripts, indicative of progenitor cell characteristics. However, this method of MaSC and progenitor cell isolation is challenging because it pushes the limits of research to identify, isolate and profile the gene signature of the harvested cells. Furthermore, identification of BrdU-LRECs with anti-BrdU antibody itself is challenging because anti-BrdU antibody only binds with BrdU antigens when the DNA is single stranded. To expose BrdU antigens in mammary cryosections, antigen retrieval using harsh chemicals, like alkali, acids or heat, is imperative. This likely destroys the morphology of the cells, as well as their nucleic acids and proteins. Additionally, the heat generated using a laser beam for microdissection will degrade RNA quality of tissue sections on glass slides [32,33]. The scant amount of nucleic acid isolated from microdissected cells was barely sufficient to perform global gene expression analysis. A novel method that permits the identification of BrdU-LRECs without compromising RNA quality [34] is reported for the laser microdissection of LRECs and non-LRECs (control cells) to enable transcriptome profiling of bovine MaSCs and progenitor cells [31]. Unfortunately, this method does not permit *in vitro* or *in vivo* analysis of the microdissected cells because the cells apoptose during harvesting. Interestingly, this method does permit study of the stem cell niche, because the cells are harvested from specific *in situ* locations.

#### Characterization of bovine and caprine MaSCs/progenitor cells

Several studies have been performed to identify bovine MaSCs and progenitor cells. Initial investigations were based on staining and morphological characteristics, namely the intensity of staining, size and shape of the cell and nucleus, nucleus to cytoplasmic ratio, and presence of cell organelles. Light stained cells were suggestive of MaSCs, which were pleomorphic and occurred singularly or in pairs [35]. Paired light stained cells were suggestive of the proliferation potential of these cells, which was later confirmed by Ki-67 expression. In pre-pubertal bovine mammary glands, approximately 10% of the epithelial cells displayed light staining, of which 50% were proliferating (Ki-67 positive).

Multiparameter cell sorting using a cocktail of antibodies appeared to be the most common method to identify MaSCs and progenitor cells in human, mice and bovine tissues. Expression of cluster of differentiation (CD) molecules, like CD24 (heat stable antigen) and CD49f (integrin alpha 6) on Lin- sorted cells, revealed features of bovine MaSCs (CD24^med^, CD49f^pos^), basal bipotent progenitors (CD24^neg^, CD49f^pos^), luminal unipotent progenitors (CD24^high^, CD49f^neg^), and luminal unipotent cells (CD24^med^, CD49f^neg^) [27].

Stem cell antigen 1 (Sca-1) appears to be a controversial marker for MaSCs. Sca-1 is a glycosyl phosphatidylinositol (GPI)-anchored cell surface protein present in the lipid raft of the cell membrane and regulates many signaling events [36]. For identification of putative bovine MaSCs in one study, Sca-1 sorted cells appeared to be located in the stroma and elicited hematopoietic transcriptomic characteristics [37]. However, MaSCs are epithelial in origin and should be localized within the epithelial compartment of the bovine mammary gland. A combination of Sca-1 marker with a panel of existing MaSC markers should enable an enriched stem cell population to be distinguished with respect to the unipotent, bipotent and truly differentiated cells. A recent study of murine mammary glands indicated that the differential gene expression profile of sorted and non-sorted cells using Sca-1, CD24 and CD49f, identified two types of luminal cells (Sca-1^pos^ and Sca-1^neg^ cells, both CD24^high^), basal cells (Sca1^neg^ CD24^low^ CD49f^high^) and myoepithelial cells (Sca1^neg^ CD24^low^ CD49f^low^) [26]. Basal cells with high CD49f expression were considered as putative MaSCs located in the basal layer.

Although fluorescent activated cell sorting (FACS) can be used to identify MaSCs and progenitor cells in various species, it has failed to provide information about the stem cell niche. This is because the preparation of a single cell suspension of mammary tissue involves enzymatic digestion of tissues and therefore disrupts all cellular and extracellular attachment of prospective stem cells. An alternative approach, BrdU-label retention method, successfully identified LRECs as enriched population of MaSC and progenitor cells [31]. LRECs had low expression of estrogen receptor (ESR1) and high expression of aldehyde dehydrogenase 3B1 (*ALDH3B1)* in the basal LRECs. Higher expression of nuclear receptor subfamily 5, group A, member 2 (*NR5A2),* a pluripotency transcription factor [38] and little to no expression of XIST, X-chromosome inactivation factor [39] in basal LREC is consistent with stem cell characteristics [31]. In the same study, embedded LRECs appeared to be more committed progenitor cells, evidenced by down-regulation of cell survival and proliferation factors *IGF2, HSPB6*, *NR5A2*, and nestin. Nestin is a mammary stem cells marker [40].

The discovery of pluripotency factors, including OCT4, SOX2 and NANOG, as new markers for MaSCs is novel [41,42]. Furthermore, the presence of MaSCs in milk and the fact that milk is a cheap and non-invasive source of MaSCs is of considerable interest [43]. However, use of pluripotency factors as additional markers of MaSC and progenitor cells remains to be validated. Understanding MaSC plasticity and the interactions between stem cells, progenitor cells, differentiated cells and stroma, is important to comprehend their biology and regulation within the gland. This will allow for the development of an effective strategy for improving milk production and livestock management.

The first demonstration of different cell types within goat mammary tissue reported the existence of luminal and myoepithelial cells [44], which were based on expression of cytokeratins (CK). Further, analyses suggested there were certain cells that were undifferentiated (observed by loss of CK expression) which remained in the alveoli of the lactating goat mammary gland. These undifferentiated cells with loss of CK, including the luminal epithelial cell differentiation marker, CK18, which indicated the presence of mammary stem or progenitor cells in the goat mammary tissue. Convincingly, the presence of caprine MaSCs and progenitor cells was demonstrated based upon *in vivo* transplantation of sorted cells in NOD/SCID mice and *in vitro* by the colony formation assay [14].

### Methods of MaSCs/progenitor cell expansion

**Figure 1:**
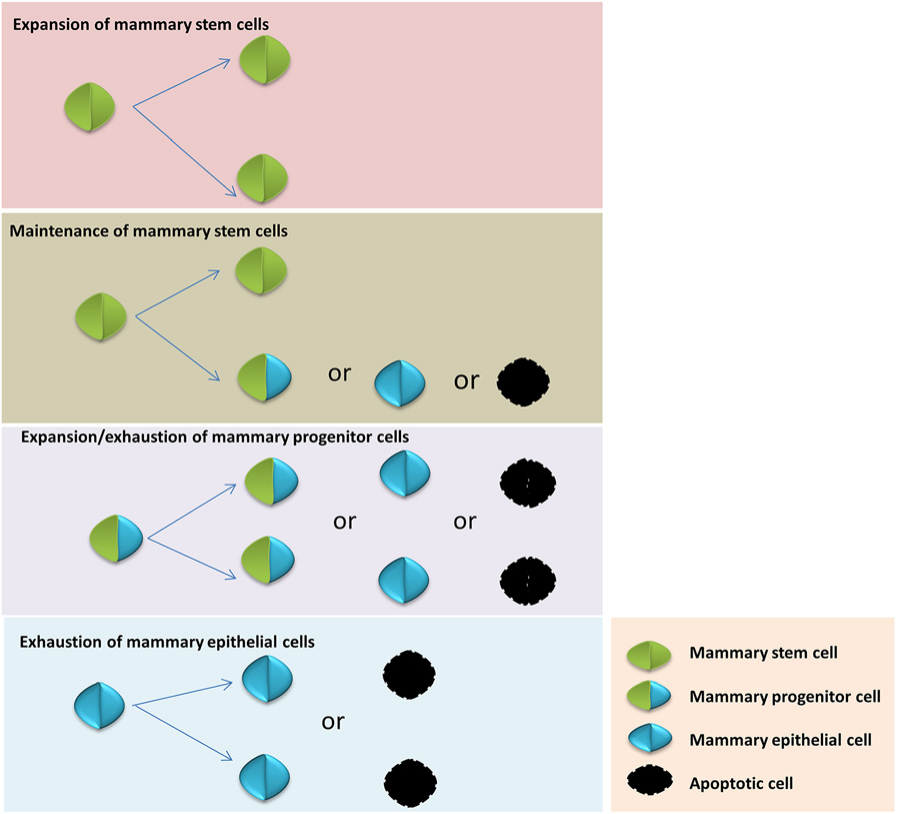
**Mammary stem cells/progenitor cells have three basic cellular division fates depending upon the physiological stage of the animal. 1)** Expansion occurs when cells divide symmetrically to produce two daughter stem cells of similar potency; **2)** Maintenance occurs when cells divide asymmetrically and produce one daughter stem/progenitor cell and one differentiated cell that may later undergo apoptosis; **3)** Expansion occurs when cells divide symmetrically, but exhaust in the case of terminal differentiation which produces two differentiated cells, both of which may later undergo apoptosis; **4)** Cells exhaust when they undergo apoptosis.

**Figure 2:**
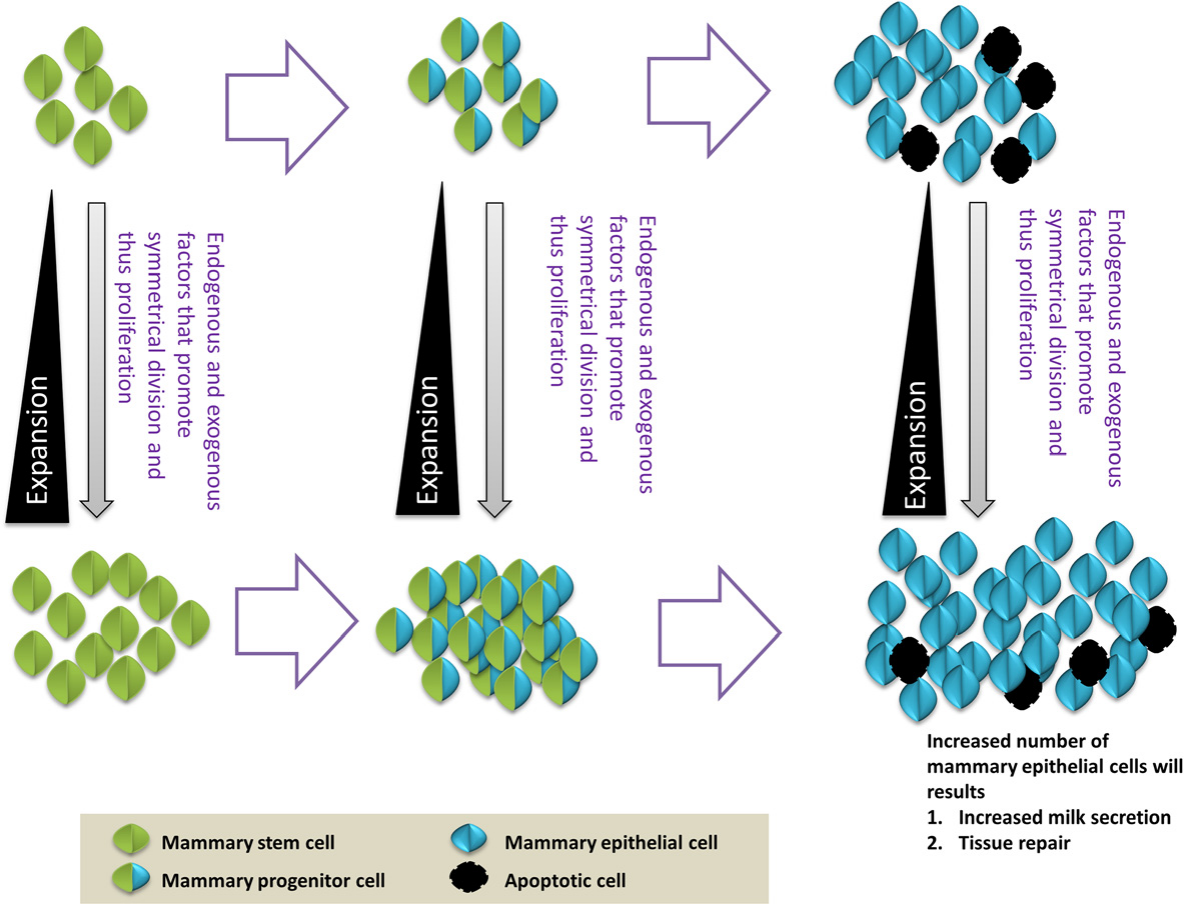
**Increased proliferation of MaSCs can increase the progenitor cell population.** Unlimited but high proliferation capacity of progenitor cells can ultimately lead to increased numbers of mammary epithelial cells. These changes in turn lead to increased secretion of milk and repair tissue damage.

The idea of MaSC expansion to increase cell turn over, enhance tissue regeneration and secretory activity of mammary epithelial cells was proposed by Capuco *et al.*[10,45,46]. During early postnatal life, increased activity of MaSCs and progenitor cells is responsible for ductal growth [46,47], which later during established lactation declines [10,48]. Stem and progenitor cells have three different fates; 1) they divide symmetrically and increase their numbers, 2) they divide asymmetrically and maintain their numbers and 3) they differentiate into terminally differentiated cells and die via programmed cell death or apoptosis (Figure 1). Endogenous factors, including the estrogen, progesterone and growth hormones, as well as exogenous compounds including bST, xanthosine and inosine, have been shown to expand MaSC and progenitor cell numbers and mammary epithelial cell populations [7,9,49-53]. It appears that these factors and compounds affect cell kinetics and enhance proliferation (Figure 2). Another instance that influences the rate of stem and progenitor cell activity is the dynamics of mammary gland physiology. An increased progenitor cell population during pregnancy indicates a role of progesterone in influencing stem and progenitor cell activity. In mice, parity- based (pregnancy associated) progenitor cells, also termed ‘parity induced MaSCs (PI-MaSCs) are reported to be located in the terminal duct of the alveolar unit, and are thought to originate from cells that skipped apoptosis during the last pregnancy [54]. The presence of PI-MaSCs has been confirmed in multiparous mice (absent in nulliparous) as multipotent stem cells by a transplantation study [55]. However, this was later refuted by generation of a mammosphere from tissue explants obtained from nulliparous mice [56]. Multiparous animals have a greater number of PI-MaSCs in the luminal epithelium than nulliparous mice [57], which is consistent with the previous study [58]. This suggests that expression of novel markers of bovine MaSCs and progenitor cells, including NUP153, NR5A2 and HNF4A [31], were significantly increased in multiparous lactating animals (peak lactation) than in nulliparous (heifer) animals. This is consistent with the idea that multiparous animals have greater numbers of PI-MaSCs than nulliparous animals. However, it remains unknown whether MaSCs and PI-MaSCs are similar or different.Taken together, these studies indicate that the physiology of the animals affects the number and activity of MaSCs/progenitor cells.

#### Use of nucleosides xanthosine and inosine

Xanthosine and inosine are purine nucleosides that act as precursors of *de novo* biosynthesis of guanine ribonucleotide. Sherley and colleagues [59,60] demonstrated that p53 mediates asymmetric division of rat hepatic stem cells and hair follicle stem cells. This action is mediated via down-regulation of inosine-5′-monophosphate dehydrogenase (IMPDH), the rate-limiting enzyme for guanine ribonucleotide synthesis. Addition of xanthosine or inosine into the system circumvents the IMPDH-mediated step and thus increases guanine concentration in the cell, thereby promoting symmetric division of stem cells and their expansion [53]. Xanthosine has been used successfully to increase stem cells, including LRECs, within the mammary gland of heifers *in vivo*[9]. Capuco and colleagues [52] further provided evidence that xanthosine increases mammary epithelial cell proliferation and the putative stem cell population in primary cultures of lactating bovine mammary epithelial cells. Details of the transcriptomic changes induced by xanthosine and the molecular mechanisms of how xanthosine alters cell proliferation, awaits further elucidation.

#### Use of growth hormone and estrogen

The scarcity of MaSCs and a universal marker to identify their pure population has hampered their study. Investigators have realized that during puberty, there is ductal growth of the mammary tree in the presence of estrogen and growth hormone. Therefore, estrogen and growth hormone might be responsible for the proliferation of MaSCs/progenitor cells present in the ducts [61]. Growth of Sca-1^pos^ cells in the presence of estrogen and growth hormone resulted in a greater number of Sca-1^pos^ cells in culture, evidenced by growth of the mammospheres and differentiation potential [62]. This study suggests that MaSC and progenitor cell populations could be increased when cells are grown in the presence of estrogen and growth hormone.

#### Use of progesterone and progestin

Progesterone is a hormone, which maintains pregnancy of the animal. One study provided the evidence of natural progesterone triggers mammary alveologenesis and expansion of MaSCs (CD24^pos^CD49f^high^) in mice [49], which was consistent with the finding that progesterone increases DNA replication and progenitor cell population in the breast [63]. These studies indicate that progesterone certainly has a role in the regulation of MaSCs/progenitor cells. In the mammary system, progesterone acts on MaSCs in a paracrine fashion [64]. Immunohistochemical analysis of bovine mammary tissue revealed PR expression in the nuclei of mammary epithelial cells, stromal and endothelial cells in heifers and lactating animals [65]. Mammary epithelial cells of the basal layer, the hypothesized location of MaSCs, usually lacks PR expression [28,65]. WNT4 and RANKL pathways mediate the mitogenic effect of progesterone [49,66]. Increased expression of RANKL in luminal cells and RANK (the receptor of RANKL) in basal cells, are the likely effectors of progesterone in basal MaSCs. Progesterone in combination with estrogen resulted in higher cell proliferation in the mammary gland than estrogen alone [67].

### Xanthosine, inosine and lactation persistency

Xanthosine has been shown to increase mammary epithelial cell proliferation [9,52]. Changes in mammary epithelial cell dynamics during lactation affects milk production. For instance, increased secretory activity of the epithelium is reported to be responsible for increased milk production from early lactation to peak lactation in cows. However a decline in milk production from peak lactation to late lactation is due to a decline in epithelial cell number [7]. Although secretory activity per cell did not change significantly from peak to late lactation, the number of secretory cells declined due to increased apoptosis in non-pregnant cows, which was responsible for the declining milk production. In pregnant and lactating cows, the effect of declining milk production was more pronounced owing to concomitant demands of nutrients for pregnancy and lactation. A reduction in milk yield was evident in continuously milked dairy cows [68-71], unlike that of goats where continuous milking did not adversely affect milk production [2,72]. In goats, continuously milked glands had a greater number of fully differentiated cells (maximum secretory activity per cell) but with fewer alveoli and thus a reduced number of mammary epithelial cells [2]. The rate of proliferation and epithelial cell differentiation also varies depending on the parity of animals. Primiparous goats are more persistent owing to higher cell proliferation and cell survival after parturition than multiparous animals [73]. If xanthosine increases mammary epithelial cell proliferation, than it would diminish cell apoptosis during late lactation. A diminished rate of cell apoptosis during late lactation will likely lead to increased availability of secretory cells (flatten the milking curve), thereby maintaining the milk production for an extended period. In other words, xanthosine treatment could be used to extend persistency of lactation.

Inosine, a compound similar to xanthosine, has been successfully used to increase milk production in transgenic goats [51]. Transgenic goats are poor milk producers owing to accelerated cell death [74] and intramammary administration of inosine during early lactation has been shown to increase milk production from day one to peak lactation period (50 days). This study was based on the experiment that demonstrated xanthosine increased MaSCs and progenitor cells in heifers [9]. This study further tested the hypothesis that stimulating MaSCs using inosine could induce the cascade of cell proliferation in transgenic goats and prevent premature cessation of lactation. Clearly, the study indicates the role of inosine in increasing MaSC numbers in transgenic goats. It has been well- documented that increased concentration of guanine ribonucleotides in stem cells favor symmetric mitotic cell division [53]. An increased number of epithelial cells, due to increased MaSCs, could have resulted increased secretory cells. An increased number of mammary secretory cells have produced more milk for extended time. It is imperative here to validate this result, that inosine really, increases MaSC number. Additionally, dosage, frequency and time of inosine administration to goats and other dairy animals need to be evaluated.

### Management of the dry period to ensure more milk production during next lactation

The non-lactating period between two consecutive lactation cycles is called the dry period. The dry period is critical to regenerate mammary epithelial cells. It is the time to replace the senescent cells that have lost production ability, with new epithelial cells that can be used to produce milk during next lactation. It can be hypothesize that during the dry period, progenitor cell activity is high which is responsible for increased cell turn over. Usually animals during the dry period are pregnant and therefore influenced by progesterone hormone, because hormone maintains pregnancy. Progesterone harbingers the mammogenic effects that are manifested by increased cell turnover and MaSC/progenitor cell activity. Usually, the length of the dry period in the cow is 50–60 days. A reduction in the non-productive dry period length is indirectly associated with increased milk production due to the reduced time of the non-productive period. At the end of the dry period lactation starts. Lactation cycle is divided into various stages- early, peak and late stage- depending upon the amount of milk produced by the animals. Maintaining the peak stage of milk production longer than average milk production is called persistency of lactation and such animals are called persistent. These persistent animals are less exposed to calving-related stress and low milk production potential during the initial lactation cycle. Apart from this, reducing the length of the dry period from 60 to 30 days could be another approach to increase the efficiency of lactation. It has been shown that a shortened dry period (30 days) or omitted dry period in the presence of bST hormone, did not alter milk production of multiparous cows during the next lactation [70]. This result was consistent with the finding that administration of bST in lactating Holstein cow increased mammary epithelial cell renewal, as evidenced by a 3-fold increase in expression of the proliferation marker, Ki-67, in bST-treated animal compared with control animals [7]. In other words, a 60-day dry period could be reduced to 30-day for regeneration of mammary epithelium without deleterious effect on milk production in next lactation.

### Conclusions and future directions

This review describes manipulation methods of MaSCs/progenitor cells that could influence future milk production, mainly in dairy animals. Emphasis has been given to cows and goat mammary glands with some imperative missing information supplemented from mice. Proliferation of both MaSCs and progenitor cells with natural hormonal treatments like progesterone, estrogen and growth hormone, and by exogenous administration of xanthosine or inosine, could potentially increase milk production of dairy animals by increasing cell turnover or persistency of lactation. Further investigations are essential to understand the biology of MaSCs/progenitor cells and their role in mammary gland morphogenesis, tissue turnover and homeostasis. Recent reports of the existence of MaSCs in breast milk and pluripotency factors as additional markers of MaSC [43], raises many questions like whether MaSCs possess multi-lineage potential. Why MaSCs are present in the milk? Does MaSCs have any role in infants who drink mother’s milk? It would also be useful to determine whether these pluripotency transcription factors are involved in the self-renewal of MaSC? Use of milk as a non-invasive source of MaSCs for their identification and characterization is a novel and promising.

## Abbreviations

MaSCs: Mammary stem cells; PI-MaSCs: Parity-induced mammary stem cells; PR: Progesterone receptor; CK: Cytokeratin; BrdU: Bromodeoxyuridine; LREC: Label-retaining epithelial cells; SE: Standard error; ESR1: Estrogen receptor; IGF2: Insulin like growth factor 2; HSPB6: Heat shock protein beta 6; OCT4: Octamer-binding transcription factor 4 (also called as POU5F1); SOX2: SRY (sex determining region Y)-box 2; NANOG: Nanog homeobox; NUP153: Nucleoporin 153; HNF4A: Hepatocyte nuclear factor 4 alpha; RANK: Receptor activator of nuclear factor κ B; bST: Bovine somatotrophin.

## Competing interests

It is declared that the author has no competing interests.

## Author’s contributions

RKC collected the information, drafted and finalized the manuscript.

## Author’s information

Ratan K. Choudhary, Ph.D., is an Assistant Professor at the School of Animal Biotechnology at Guru Angad Dev Veterinary and Animal Science University, Ludhiana, Punjab, India. The author has been working with bovine mammary stem cells, their molecular characterization and expansion for the last seven years.
